# Attitudes towards sexual education among midwifery students

**DOI:** 10.18332/ejm/172511

**Published:** 2023-11-08

**Authors:** Emel Bahadır Yılmaz

**Affiliations:** 1 Faculty of Health Sciences, Giresun University, Giresun, Turkey

**Keywords:** sexual education, midwifery student, midwifery care

**Dear Editor,**

Midwifery students have an essential role in improving the sexual health of individuals, families, and communities^[Bibr cit0001]1^. Studies conducted with midwifery students show that students are inadequate in sexual education and counseling^2,3^. However, sexual health counseling given by midwives increased women’s sexual satisfaction during pregnancy and postpartum, decreased their inefficient sexual beliefs, and increased the sexual function of postmenopausal women^4-6^. Midwifery students need to have positive attitudes towards sexuality to be able to provide sexual health counseling in their professional life as midwives. Therefore, this cross-sectional study aimed to determine midwifery students’ attitudes toward sexual education.

This study was conducted during the 2018–2019 academic year. The study sample consisted of 235 midwifery students in the Faculty of Health Sciences at a state university in northeastern Turkey. Students completed two questionnaires: the Student Information Form and the Inventory of the Attitude toward Sexual Education^7^. Written permission was obtained from the university ethics committee (Date of Decision: 25/03/2019 Decision No: 2019/24) and by the Faculty of Health Sciences. The study was performed following the Declaration of Helsinki. Data were analyzed using the SPSS 24 package program. Descriptive statistics, Kolmogorov–Smirnov test, Mann Whitney U test, and Kruskal Wallis test, were used in data analysis.

The mean age of the students participating in the study was 20.65 ± 2.04 years (range: 18–38). The majority of the respondents were single, 72.3% had a nuclear family structure, 48.9% lived in a city, 87.7% had moderate socioeconomic status, and the incomes of 76.2% were equal to their expenses. Of the students, 20.4% received sex education from their families; 34.9% received sex education before college; 40.4% received sex education at university; 79.6% wanted to learn more about sexuality; and 36.2% evaluated the patients’ sexual life in the clinic. There was no significant difference between the scale scores according to classes, sex education status, and evaluation of the patients’ sexual life in the clinic (p>0.05). However, perceived socioeconomic status, mother’s education level, and desire to receive education about sexuality affected students’ attitudes (p<0.05).

In conclusion, we can say that there is a gap in the midwifery curriculum related to sex education in this study, because the number of students who do not receive sex education is high, and there is no difference between the attitudes toward sex education of students who do and do not receive sex education. The most critical barriers for midwifery students were lack of experience with sex medicine, fear of failing to respond to patients’ sexual health issues, and lack of knowledge^8^. Midwifery care includes counseling on sexual health, and it is a fundamental human right and an important step in enabling women to control their health outcomes^9^. Interactive workshops with standardized patients can be held to increase sex knowledge and skills of midwifery students^10^. In clinical practices, the students should assess sexuality in midwifery care and receive mentoring on this subject.

## Data Availability

Data sharing is not applicable to this article as no new data were created.
